# Feasibility and Acceptability of Internet Grocery Service in an Urban Food Desert, Chicago, 2011-2012

**DOI:** 10.5888/pcd10.120299

**Published:** 2013-05-02

**Authors:** Bradley M. Appelhans, Elizabeth B. Lynch, Molly A. Martin, Lisa M. Nackers, Vernon Cail, Nicole Woodrick

**Affiliations:** Author Affiliations: Elizabeth B. Lynch, Molly A. Martin, Lisa M. Nackers, Vernon Cail, Nicole Woodrick, Rush University Medical Center, Chicago, Illinois.

## Abstract

We explored the feasibility and acceptability of an Internet grocery service (IGS) as an approach to improving food access in urban neighborhoods. In our pilot study, caregivers residing in a documented Chicago food desert (N = 34, 79% ethnic minority) received a voucher to use a commercial IGS to purchase groceries for their household. Caregivers most frequently purchased fruits, vegetables, meats, and caloric beverages, and endorsed 4 factors as potentially important determinants of future IGS use. IGS programs could have a role in improving urban food access if they have competitive prices, provide rapid delivery, and incorporate strategies to discourage purchasing of discretionary caloric beverages.

## Objective

Proposals to improve urban food access include providing incentives for new supermarket development, facilitating transportation to existing supermarkets, and using farmers markets and street vendors as supplemental food sources (1–4). However, these approaches are costly and face logistical challenges (5,6). A novel strategy to improve food access involves Internet grocery service (IGS) that delivers healthful foods to the home. IGS is convenient, may overcome potential transportation barriers, and does not require new supermarket construction; however, it does involve delivery fees and requires Internet access. This feasibility pilot study examined IGS food purchasing patterns and acceptability among caregivers in a documented urban food desert.

## Methods

This cross-sectional study documented food purchasing patterns and assessed acceptability during a field test of a commercial IGS. Participants were caregivers of children aged 2 to 14 years who reported making most of household food purchases and who lived in a documented Chicago food desert from 2011 to 2012 (7). Exclusion criteria included having a household member with major dietary restrictions and having used IGS in the past 30 days. Recruitment methods included posted advertisements, physician referrals, and word-of-mouth. Rush University Medical Center’s institutional review board approved study procedures.

Participants completed 2 study visits. In the first visit, participants completed surveys on race/ethnicity, household income, marital status, household composition, automobile ownership, and home Internet access. Body mass index (BMI [kg/m^2^]) was calculated from height and weight measurements. Participants then used a computer at the research facility to purchase groceries through www.Peapod.com. We provided an $80 voucher for groceries and delivery fees, which ranged from $6.95 to $9.95. One participant purchased additional groceries with her own funds. Peapod.com does not accept Supplemental Food Assistance Program (SNAP) vouchers (formerly called food stamps). Participants selected a delivery time window from choices available, and returned to our offices several days after receiving delivery to complete assessments of IGS acceptability. As in other pilot studies (8), participants rated the IGS on perceived convenience, ease of use, and satisfaction on a 5-point scale: 1, very inconvenient/difficult/unsatisfied; 2, inconvenient/difficult/unsatisfied; 3, neither convenient nor inconvenient/easy nor difficult/satisfied nor dissatisfied; 4, convenient/easy/satisfied; 5, very convenient/easy/satisfied. Participants also reported how often they planned to use IGS in the future and which of 7 different factors would strongly affect their future IGS use. Participants were compensated $25 at the second visit.

Descriptive statistics characterized the sample and summarized responses to the surveys. IGS food purchases were quantified as the number of items and total pre-tax food expenditures in 11 categories. We used measures of central tendency (mean and SD) to report continuous variables and frequencies, and percentages to report categorical/count variables. Data were analyzed using Stata 11 (StataCorp, College Station, Texas).

## Results

Of 60 caregivers screened, 37 were enrolled, and 34 (77% female, 38% black/African-American, 32% Hispanic/Latino, 21% non-Hispanic white, 9% multiethnic) completed the study and were included in analyses (Peapod receipts were incomplete or unavailable for 3 subjects who completed the study). Caregivers were aged 36.9 years (SD, 9.6) with a mean BMI of 32.9 kg/m^2^ (SD, 8.0); on the basis of BMI, 13% were overweight, and 65% were obese. Households averaged 3.7 (SD, 1.7) members, with 53% of caregivers reporting their marital status as single, 29% as married or living with a partner, and 18% as divorced or widowed. Annual household income was below $40,000 for 79% of the sample. Most caregivers owned at least 1 working automobile (68%) and a working computer with home Internet access (74%).

Participants purchased an average of 23.5 items (SD, 11.1) at a total cost of $69.36 (SD, $16.08) (Table 1). The largest share of pre-tax food expenditures (36%) was for meat, fish, poultry, and egg- and dairy-based dishes. Fruits, vegetables, and caloric nondairy beverages (eg, carbonated soft drinks, juice) were also frequently purchased, whereas noncaloric beverages, sweets, desserts, and candy were purchased infrequently.

**Table 1:** Internet Grocery Service Food Purchases by Caregivers (N = 31) Residing in an Urban Food Desert, Chicago, 2011-2012

Food category	No. of Items, Mean (SD)	Pre-tax Cost, Mean (SD), $	
All	23.5 (11.1)	69.36 (16.08)
Meat, fish, poultry, egg- and dairy-based dishes	5.6 (4.5)	25.28 (18.38)
Fruits^a^	5.3 (5.9)	8.61 (8.88)
Vegetables^b^	3.6 (6.9)	6.55 (10.62)
Carbohydrates (eg, bread, cereal, crackers)	3.5 (2.8)	10.43(7.54)
Mixed dishes and soups	1.9 (2.9)	5.45 (6.95)
Caloric nondairy beverages (eg, carbonated soft drinks, juice)	1.5 (1.0)	5.99 (8.02)
Condiments	0.9 (1.5)	2.72 (5.29)
Caloric dairy beverages (eg, milk)	0.5 (0.6)	1.50 (1.92)
Candy, fruit snacks, liquid sweets	0.4 (0.8)	1.09 (2.10)
Cakes, cookies, pastries, muffins, pies	0.3 (0.6)	1.01 (2.43)
Noncaloric beverages (eg, tea, diet soft drinks)	0.1 (0.1)	0.42 (1.31)

a Includes whole fruit and nondessert, fruit-based dishes such as fruit salad.

b Includes whole vegetables and vegetable-based dishes such as bagged salads.

Participant satisfaction with various aspects of the IGS averaged between “somewhat dissatisfied” and “somewhat satisfied.” Most participants (54.5%) intended to use an IGS 1 to 6 times per year, and 18.2% reported they intended to use an IGS monthly (Table 2). Of 7 factors assessed, 2 were endorsed as strong influences on future IGS use by more than half of participants: IGS prices equal to or lower than supermarkets (79.4%), and foods delivered within 1 day of ordering (55.9%). Two other factors, ability to shop using a home computer and acceptance of SNAP vouchers as a payment method, were both endorsed by 44.1% of participants.

**Table 2:** Acceptability of and Intention to Use Internet Grocery Service (IGS) Among Caregivers Residing in an Urban Food Desert (N = 34), Chicago, 2011-2012

Factor	Value
**Acceptability^a^**	**Mean (SD)**
Convenience of home food delivery	3.6 (0.8)
Satisfaction with IGS prices	2.6 (1.0)
Satisfaction with quality of foods	3.5 (0.6)
Satisfaction with variety of foods offered	3.4 (0.8)
Web site ease of use	3.5 (1.0)
**Intention to use IGS in the future (n = 33)**	**n (%)**
Never	3 (9.1)
1–6 times per year	18 (54.5)
7–11 times per year	3 (9.1)
1 time per month	6 (18.2)
2 times per month	2 (6.1)
3 times per month	1 (3.0)
Every week, or every time I buy groceries	0
**Factors that would strongly influence future use of IGS (n = 34)^b^**	**n (%)**
Accepting Supplemental Food Assistance Program vouchers as payment	15 (44.1)
Prices equal to or lower than supermarkets	27 (79.4)
Able to shop using computers in public locations	5 (14.7)
Able to shop using a computer at home	15 (44.1)
Foods delivered within 3 days of ordering	2 (5.9)
Foods delivered within 2 days of ordering	5 (14.7)
Foods delivered within 1 day of ordering	19 (55.9)

a Scored on a 5-point scale: 1, very inconvenient/difficult/unsatisfied; 2, inconvenient/difficult/unsatisfied; 3, neither convenient nor inconvenient/easy nor difficult/satisfied nor dissatisfied; 4, convenient/easy/satisfied; 5, very convenient/easy/satisfied.

b Answered in a yes/no format, with number and percentage responding yes reported.

## Discussion

This study provides partial support for the feasibility and acceptability of IGS as a strategy to increase urban food access. Fruits and vegetables, which are commonly lacking in urban food deserts (9,10), represented a large proportion of IGS expenditures. Candy, desserts, and sweets represented a small proportion of expenditures, but many caregivers purchased caloric beverages, which contribute to obesity (11). Therefore, efforts to increase food access through IGS should be paired with programs aimed at reducing consumption of caloric beverages.

Participants were somewhat satisfied with the variety and quality of foods offered and the convenience of delivery but were somewhat dissatisfied with food prices. Our study showed that a well-implemented IGS would 1) have competitive prices, 2) deliver groceries within 1 day, 3) accept food assistance, and 4) be accessible through home Internet service.

Study strengths include the use of an IGS field test and well-defined geographic criteria to identify food desert households. Study limitations include a small sample size and a lack of a comparison with purchases from traditional food sources. Because of its cross-sectional design, our study cannot determine whether purchasing patterns would be sustained long-term. Given our focus on evaluating IGS feasibility and acceptability rather than efficacy, we provided an $80 IGS voucher to enable participation by disadvantaged participants who were unable to pay out-of-pocket to try a new food source. Though this voucher was necessary for recruitment purposes, it may have influenced purchasing patterns.

IGS programs could play an important role in improving urban food access if they are designed to overcome the barriers to use identified in this study and if they incorporate strategies to discourage purchase of caloric beverages. We encourage further developmental research using more rigorous designs and longer follow-up periods to assess the effects of IGS use on food purchasing and dietary intake.
